# Identifying X (Formerly Twitter) Posts Relevant to Dementia and COVID-19: Machine Learning Approach

**DOI:** 10.2196/49562

**Published:** 2024-06-04

**Authors:** Mehrnoosh Azizi, Ali Akbar Jamali, Raymond J Spiteri

**Affiliations:** 1 Department of Computer Science University of Saskatchewan Saskatoon, SK Canada

**Keywords:** machine learning, dementia, Alzheimer disease, COVID-19, X (Twitter), natural language processing

## Abstract

**Background:**

During the pandemic, patients with dementia were identified as a vulnerable population. X (formerly Twitter) became an important source of information for people seeking updates on COVID-19, and, therefore, identifying posts (formerly tweets) relevant to dementia can be an important support for patients with dementia and their caregivers. However, mining and coding relevant posts can be daunting due to the sheer volume and high percentage of irrelevant posts.

**Objective:**

The objective of this study was to automate the identification of posts relevant to dementia and COVID-19 using natural language processing and machine learning (ML) algorithms.

**Methods:**

We used a combination of natural language processing and ML algorithms with manually annotated posts to identify posts relevant to dementia and COVID-19. We used 3 data sets containing more than 100,000 posts and assessed the capability of various algorithms in correctly identifying relevant posts.

**Results:**

Our results showed that (pretrained) transfer learning algorithms outperformed traditional ML algorithms in identifying posts relevant to dementia and COVID-19. Among the algorithms tested, the transfer learning algorithm A Lite Bidirectional Encoder Representations from Transformers (ALBERT) achieved an accuracy of 82.92% and an area under the curve of 83.53%. ALBERT substantially outperformed the other algorithms tested, further emphasizing the superior performance of transfer learning algorithms in the classification of posts.

**Conclusions:**

Transfer learning algorithms such as ALBERT are highly effective in identifying topic-specific posts, even when trained with limited or adjacent data, highlighting their superiority over other ML algorithms and applicability to other studies involving analysis of social media posts. Such an automated approach reduces the workload of manual coding of posts and facilitates their analysis for researchers and policy makers to support patients with dementia and their caregivers and other vulnerable populations.

## Introduction

Dementia is a group of progressive syndromes that cause impairments in high-level cognitive functions, such as memory, language, and thinking, as well as everyday functioning and social interactions [1]. Currently, approximately 50 million people worldwide are affected by dementia, and this number is projected to continue to rise rapidly [2]. As populations age worldwide, dementia is expected to continue to be a significant health care challenge, requiring considerable medical, social, and institutional care [3].

The emergence of the COVID-19 pandemic presented an additional challenge for patients with dementia and their caregivers. Older patients were at a higher risk of contracting COVID-19 and were more likely to experience severe symptoms and consequences due to age, vulnerability, frailty, and other health conditions commonly associated with dementia [4]. In the United Kingdom, half (50%) of the COVID-19–related deaths in care homes were among patients with dementia [5]. Patients with dementia face challenges in adhering to self-protection protocols, such as wearing masks, using hand hygiene, and practicing physical distancing. In addition, they may have difficulty understanding and remembering the risks associated with COVID-19 [6]. In addition, the caregivers of patients with dementia also face limitations imposed by COVID-19, such as social isolation, loss of support, and care-partner burnout, all of which can further exacerbate the unpleasant situation of patients with dementia by limiting their access to public services and the support provided by their caregivers [6].

Social media platforms have emerged as valuable sources of information for individuals seeking updates on various issues, performing scientific studies, providing support, and raising public awareness. X (formerly Twitter), established in 2006 with about 400 million users, is a popular social media platform for microblogging where users can publicly share their thoughts using short messages called posts (formerly tweets). Posts can provide insight into COVID-19–related experiences of patients with dementia and their caregivers, offering an opportunity to study the impact of the pandemic on them. By identifying and analyzing posts relevant to dementia and COVID-19, substantial opportunities to support effective decision-making and policy development and further research can be uncovered.

Manual analysis, mining, and coding of posts can be a challenging, time-consuming, and laborious task, hindering the ability of health care researchers and practitioners to gain insights into the impact of COVID-19 on patients with dementia and other vulnerable populations. As such, there is a need to improve existing methodologies to streamline and automate the process. Machine learning (ML) algorithms have become a popular approach for performing tasks such as thematic or semantic analysis, and the classification of posts with reliable accuracy, offering a promising solution to this challenge [7-11].

ML algorithms have shown promise in classifying posts based on different sentiments. For example, Roy and Ojha [12] trained 3 classifiers using automatically labeled data in their study. However, the inclusion of a significant amount of noise through the automated labeling of data resulted in poor performance of their classifiers. Chiroma et al [13] manually labeled posts and used ML algorithms to classify suicide-related posts. The authors applied the Bag of Word technique for feature extraction and evaluated the performance of 5 traditional ML algorithms. Although there was no significant difference in performance among different algorithms for most classifications, binary classification showed the most promising results.

**Figure 1:**
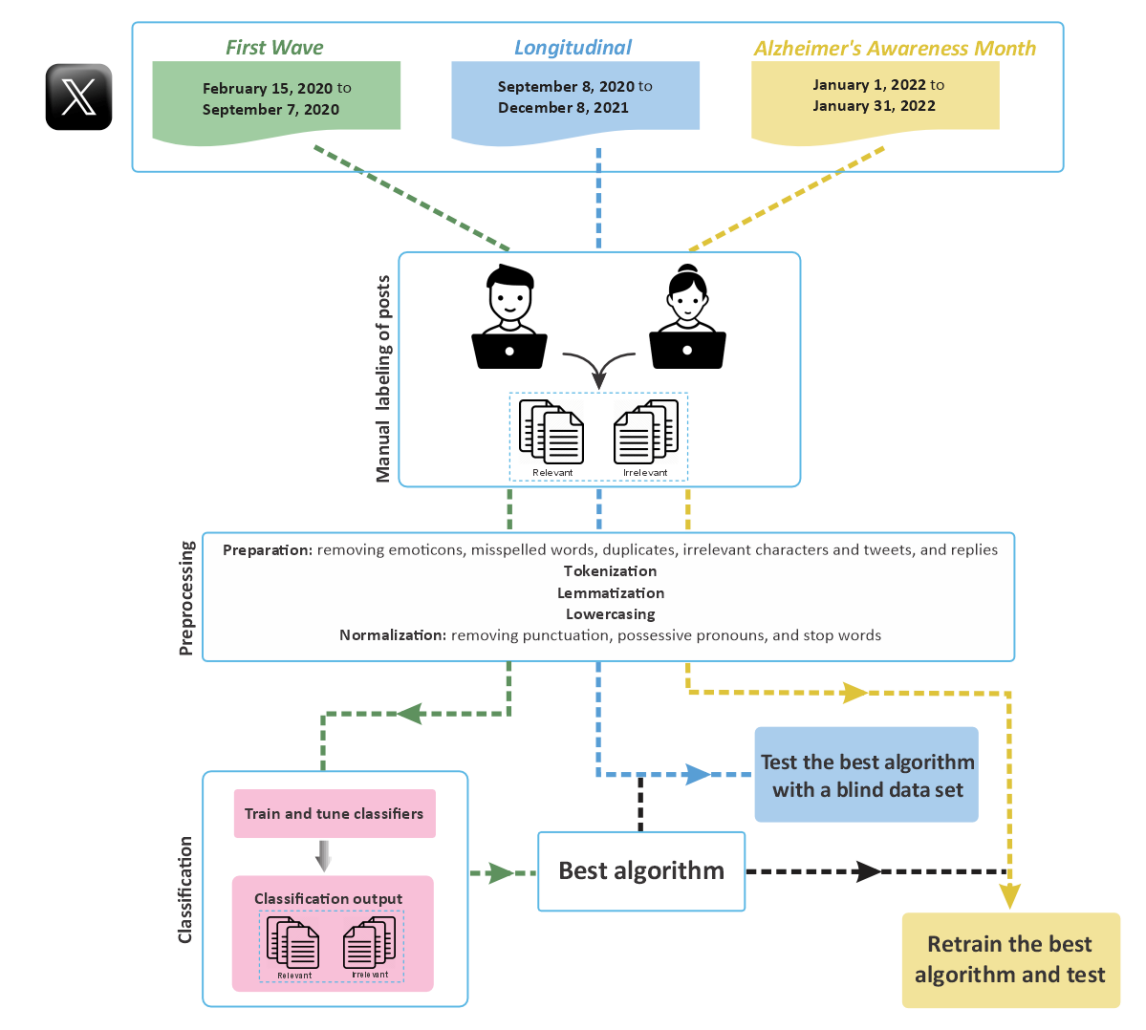
The workflow of this study. (1) Data collection involved gathering posts containing search keywords from 3 distinct data sets: the First Wave data set consisting of 5063 posts from February 15, 2020, to September 7, 2020; the Longitudinal data set consisting of 110,528 posts from September 8, 2020, to December 8, 2021; and the Alzheimer’s Awareness Month data set consisting of 1289 posts from January 1, 2022, to January 31, 2022; (2) data preprocessing, and (3) classification/evaluation.

Despite these results, recent studies have raised concerns about the effectiveness of traditional ML algorithms for identifying and classifying posts [14,15]. Therefore, this study aims to demonstrate an alternative method for identifying posts relevant to dementia and COVID-19 using a combination of natural language processing (NLP) and ML algorithms and explore the reliability and performance of these algorithms. By leveraging the power of more advanced algorithms and techniques, specifically transfer learning algorithms, we aim to develop a model that can effectively analyze large volumes of posts to identify relevant posts and potentially offer valuable insights to policy makers, health care professionals, and researchers to inform evidence-based decision-making and support patients with dementia and their caregivers. Furthermore, the methodology described applies more generally to identify relevant social media posts to help facilitate further analysis. The overview and workflow of our study are shown in Figure 1.

## Methods

### Data Collection

In this study, we aimed to analyze posts and identify those relevant to dementia and COVID-19. We used 3 data sets for this purpose. The first data set, referred to as the First Wave data set, consisted of 5063 posts collected between February 15, 2020, and September 7, 2020, using the search terms “dementia” or “Alzheimer” in combination with “COVID-19,” “COVID,” or “Corona” [16]. This data set captured the experiences of people impacted by Alzheimer disease or dementia and COVID-19 in the early stages of the pandemic. To capture the later stages of the pandemic, we collected a second data set, referred to as the longitudinal data set, consisting of 110,528 posts between September 8, 2020, and December 8, 2021, using the same search terms as the first data set. Finally, to examine the X discourse on dementia during Alzheimer’s Awareness Month in Canada, we collected a third data set, referred to as the Alzheimer’s Awareness Month data set, comprising 1289 posts between January 1, 2022, and January 31, 2022. Table 1 summarizes the particulars of the data sets, including the number of instances, and search terms for each data set. Figure S1 in Multimedia Appendix 1 depicts the word cloud of the most frequently used word in each data set.

**Table 1:** The characteristics of different data sets^a^.

Data set	Posts, n	Time frame	First search terms	Second search terms
First Wave	5063	February 15, 2020, to September 7, 2020	Dementia, Alzheimer	COVID-19, COVID, Corona
Longitudinal	110,528	September 8, 2020, to December 8, 2021	Dementia, Alzheimer	COVID-19, COVID, Corona
Alzheimer’s Awareness Month	1289	January 1, 2022, to January 31, 2022	#AlzheimersAwarenessMonth, #AlzAwareness, #dementiawareness, dementia month, dementia awareness month, Alzheimer’s awareness month, Alzheimer’s month, January is Alzheimer’s Awareness month	@alzCanada, @AlzheimerOnt, @AlzheimerSK, @DementiaAB\_NT, @AlzheimerNS, @AlzheimerNB, @AlzheimerPEI, @alzheimerMB, @AlzheimerBC, @asnl2, @FqsaAlzh

^a^The data applied in the study consist of 3 distinct data sets including different search keywords.

### Data Preprocessing

To construct data sets consistently, preprocessing was performed on all 3 data sets to remove noise. Preparation steps such as simple filtering (removing emoticons, misspelled words, duplicates, obviously irrelevant characters, posts, and replies), tokenization, lemmatization, lowercasing, and normalization (removing punctuation, possessive pronouns, and stop words) were applied. After this filtering, the remaining posts were manually labeled as either relevant or irrelevant by a team of 11 coders using thematic analysis and the codebook from Bacsu et al [16]. Intercoder reliability was assessed through random reviewing of 25% (1560/6243) of all labeled posts and an average intercoder reliability of 83.4% was achieved.

### Feature Extraction

After the preprocessing steps, the tokenized words were then converted into vectors using the term frequency–inverse document frequency (TF-IDF) method [17-19]. TF-IDF calculates the relative frequency of words in a specific document compared with the inverse proportion of that word over the entire text document determining the importance of a given word in a document. Accordingly, words frequently used in a document have higher TF-IDF weights and are regarded as more representative. Words common to many documents, such as articles and prepositions, have lower TF-IDF weights and are considered less representative [18,19]. The TF-IDF hyperparameter, max-features, determines the number of the most representative words for the rest of the analysis [18]. We set the max-features threshold to 1250 based on experimentation and the scores of different ML classifiers. This approach helps select the most representative words for the highest performance.

### ML Algorithms

#### Overview

Various traditional and transfer learning ML algorithms were applied to identify posts relevant to dementia and COVID-19.

#### Traditional ML Algorithms

##### Logistic Regression

Logistic regression (LR) is a statistical ML method used for analyzing the relationship between a binary dependent variable and 1 or more independent variables. It is commonly used for classification tasks, where the goal is to predict the probability of an event occurring based on a set of input variables. The output of a logistic regression model is a probability value between 0 and 1, which can be interpreted as the likelihood of the event occurring [20].

##### Naïve Bayes

Naïve Bayes (NB) is a probabilistic ML algorithm that is based on the Bayes rule. It uses the assumptions of strong independence between variables to construct a simple and fast algorithm [21].

##### Multinomial Naïve Bayes

Multinomial Naïve Bayes (MNB) is a probabilistic ML algorithm based on Bayes’ theorem that calculates the probability of a particular event occurring based on prior knowledge of the conditions that may affect that event [22,23]. In text classification, the algorithm learns the probability distribution of words in each class and then calculates the conditional probability of a new document belonging to each class given its word frequency distribution.

##### K-Nearest Neighbors

K-nearest neighbors (kNN) is a nonparametric ML algorithm known for its simplicity and effectiveness. kNN classifies data based on the closeness of training samples in a given region [24].

##### Support Vector Machine

Support vector machine (SVM) is a widely used supervised ML algorithm for classification and regression analysis. SVMs are based on finding the best hyperplane that separates data into different classes. The hyperplane is chosen such that it maximizes the distance between the 2 classes [25].

##### Decision Tree

Decision tree (DT) is a supervised ML algorithm that is designed to solve classification problems by learning a hierarchy of “if/else” questions and answers and by creating a tree representation that results in a decision [26]. The goal of the DT classifier is to get the right classification result by asking the least number of “if/else” questions.

### Ensemble Algorithms

#### Overview

Ensemble algorithms are designed to improve the accuracy of classification by combining multiple base classifiers. Although any type of base classifier including DT, neural networks, or SVMs can be used to create ensemble algorithms, DT is a commonly used algorithm [27].

#### Random Forest

Random forest (RF) is a powerful ensemble algorithm that uses DTs as the base classifiers. In RF, a set of features is randomly selected to determine the best split at each node of the DT. To make a prediction for a new data point, RF first applies each DT in the forest and predicts the target. Then, it uses the majority vote of all the DT predictions to assign the target with the highest probability to the new data [27,28].

#### AdaBoost

Adaboost (Adaptive Boosting) is a boosting algorithm that combines weak classifiers to create a strong classifier. It works by iteratively adjusting the weights of misclassified samples and adding new weak classifiers based on the samples that were misclassified in the previous iteration. This makes the algorithm adaptive, enabling it to focus on the more difficult samples in subsequent iterations. The final classification is determined by a weighted combination of the weak classifiers [29].

#### XGBoost

Extreme Gradient Boosting (XGBoost) is a distributed boosting algorithm that uses regression trees as a base classifier. It is designed to improve the accuracy of gradient boosting and is known for its high predictive power and speed, capable of being many times faster than other boosting techniques due to its parallel and distributed computing capabilities [30]. In addition, XGBoost performs well in sparse feature spaces [31] and uses more accurate approximations to find the best tree model.

### Transfer Learning Algorithms

#### Overview

Transfer learning refers to transferring knowledge from different but related source domains to the target model in target domains in order to improve the performance of the target model. Consequently, a target model can be constructed without having to rely on a large number of domain data [11,32].

#### Bidirectional Encoder Representations From Transformers

Bidirectional Encoder Representations from Transformers (BERT) is a transfer learning algorithm that uses bidirectional transformers to create contextualized embeddings for each word in a sentence or text. It has achieved state-of-the-art performance in a wide range of NLP tasks, including text classification, question answering, and language understanding [33]. BERT has 2 phases: pretraining and fine-tuning. In the pretraining phase, the model is trained on a large corpus of text to learn general language patterns. In the fine-tuning phase, the pretrained model is adapted to a specific task by training it on a smaller, task-specific data set [34].

#### A Lite BERT

A Lite BERT (ALBERT) is a variant of BERT that reduces the number of parameters in the model while maintaining the same performance. This is achieved by applying 2 parameter reduction techniques: factorized embedding parameterization and cross-layer parameter sharing. ALBERT has been shown to have faster training times and better scalability than BERT, making it a useful option for large-scale NLP tasks [35].

### Evaluation

To evaluate the performance of compared algorithms, we randomly partitioned the First Wave data set into 2 subsets: a training set used for cross-validation (CV; 4050/5063, 80% labeled posts) and a test set (1013/5063, 20% labeled posts). The CV set was further divided into 10 subsets, allowing us to construct and train 10 distinct models. The CV set was further partitioned into 10 subsets, enabling the construction and training of 10 distinct models. Using unseen test data is essential to evaluate the models’ generalization capability. This assessment with unseen data minimizes the risk of overfitting and offers a more dependable estimate of the algorithm’s real-world performance. Then, we selected the best-performing classifier and evaluated its reliability on unseen data using the longitudinal data set. A reliable algorithm should accurately predict the class of a large portion of the unseen data. Finally, we trained and tested the best-performing algorithm on the Alzheimer’s Awareness Month data set, using a training set (129/1289, 10% labeled posts) and a test set (1160/1289, 90% labeled posts). To assess the performance of the used ML algorithms, evaluation metrics including accuracy, precision, sensitivity, specificity, *F*_1_-score, and area under the curve (AUC) were used. These metrics provide a comprehensive understanding of the performance of algorithms in terms of correctly identifying relevant posts and minimizing false positives and false negatives.

### Parameter Sensitivity Analysis

To optimize the performance of competing algorithms, it is necessary to tune their parameters. However, there is no one-size-fits-all approach for parameter selection. Therefore, in this study, we explored different sets of parameter values for each algorithm to identify the optimal configuration. A summary of the ML algorithms and their respective parameters used in this study can be found in Table 2.

**Table 2:** Parameters of different ML^a^ algorithms^b^.

Algorithm	Parameters
LR^c^	C: [0.1, 0.5, 1,2] Multiclass: [ovr] Solver: [Newton-CG, IBFGS, Liblinear, SAG, SAGA]
kNN^d^	Number of neighbors: [3, 5, 7, …, 51] Metrics: [Euclidian, Manhattan]
SVM^e^	C: [0.1, 0.5, 1, 1.5, 2] Kernel: [linear, poly, RBF, sigmoid]
DT^f^	Criterion: [Entropy, Gini] Min sample leaf: [1,2,4,6,8] Min sample split: [1, 2, 3, …, 10, …, 20]
RF^g^	N estimators: [100, 200, 300, 400, 500] Criterion: [Entropy, Gini] Max depth: [2, 4, 6, …, 32, …, 64]
Adaboost^h^	Base estimators: [LR, DT, SVM] N estimators: [10, 20, 30, …100, …, 500] Max depth: [1, 2, 3, 4, …, 20]
XGBoost^i^	Max depth: [1, 2, 3, 4, …, 19, 20] Learning rate: [0.01, 0.015, 0.02, 0.025, …, 0.1]

^a^ML: machine learning.

^b^Each algorithm has different parameters. The values in brackets represent values for each parameter.

^c^LR: logistic regression.

^d^KNN: k-nearest neighbor.

^e^SVM: support vector machine.

^f^DT: decision tree.

^g^RF: random forest.

^h^Adaboost: Adaptive Boosting.

^i^XGBoost: Extreme Gradient Boosting.

### Implementation

Posts were obtained using TWINT, a powerful scraping tool that allows for the scraping of posts without the requirement for an X account and the use of X’s application programming interface, enhancing the number of posts scraped, frequency, and time period of scrapes. We used Python programming language (version 3.8.5; Python Software Foundation) using NLTK [36], Scikit-Learn [37], and TensorFlow [38] libraries. The computations were performed on an Nvidia Tesla T4 GPU (Nvidia Corporation) within Google Colab.

### Ethical Considerations

Ethical considerations about social media research suggest that publicly available data (eg, posts on X) can be used for research studies without requiring additional consent or ethics approval [39]. In this study, we did not apply for the ethics approval. In addition, because we did not engage or interfere with the users whose publicly posted content was collected and analyzed, we did not require informed consent. Nonetheless, to ensure users’ anonymity and protect their privacy, any related identifying personal information (eg, user IDs and usernames) has been removed. There was no compensation provided to the users whose public X posts were used.

## Results

### Parameter Sensitivity Analysis

In this study, several algorithms were used with multiple parameters that were tuned to obtain optimal performance. AdaBoost performed best with a base estimator of DT, N-estimator set to 100, and a max depth set to 5. DT achieved the best performance with the criterion parameter set to “entropy,” a minimum sample leaf set to 100 and a minimum sample split set to 5. KNN performed best with 5 neighbors and with the “Euclidean” distance metric. LR achieved the best performance with a parameter *c* of 0.5, “ovr” selected for multiclass and “liblinear” selected for solver parameters. RF performed best with “Gini” as the criterion parameter, N-estimator set to 300, and max depth set to 32. SVM performed best with a *radial basis function* kernel and *C* set to 5. Finally, XGBoost performed best when max depth and learning rate were set to 6 and 0.015, respectively.

### Performance Analysis

#### Study 1

The performance of various algorithms in identifying posts relevant to dementia and COVID-19 was evaluated with the First Wave data set. SVM achieved the highest accuracy (96.17%), precision (93.08%), sensitivity (98.04%), specificity (94.86%), *F*_1_-score (95.49%), and AUC (99.41%) for the training set, followed by the RF algorithm obtaining the second-best performance across all metrics. Table 3 summarizes the performance metrics of different algorithms for the training set.

**Figure 2:**
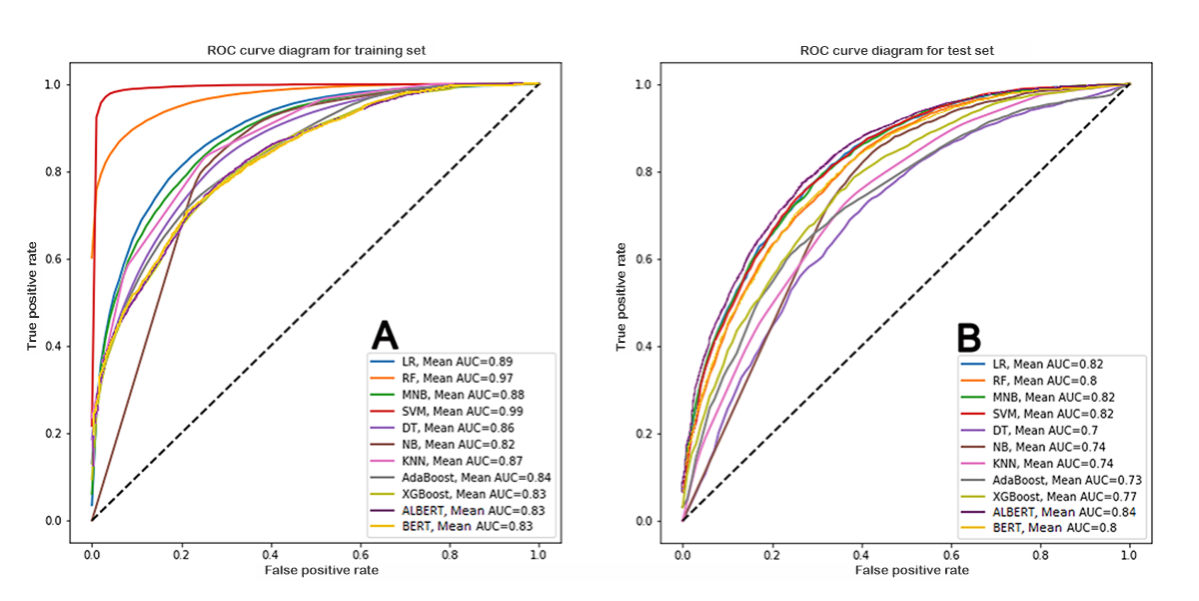
Receiver operating characteristic curves for competing machine learning algorithms for training (A) and test sets (B). The corresponding area under the curve values are given for each algorithm. ROC: receiver operating characteristic; LR: logistics regression; AUC: area under the curve; NB: Naïve Bayes; MNB: Multinomial Naïve Bayes; kNN: k-nearest neighbor; SVM: support vector machine; DT: decision tree; RF: random forest; BERT: Bidirectional Encoder Representations from Transformers; ALBERT: A Lite BERT.

For the test set, the ALBERT algorithm achieved the best accuracy (82.92%), precision (74.55%), sensitivity (84.83%), specificity (82.24%), *F*_1_-score (78.81%), and AUC (83.53%) among all the algorithms tested. The BERT algorithm achieved the second-best performance for these metrics. Performance metrics of different algorithms for the test set are summarized in Table 4, and the receiver operating characteristic curves of competing algorithms for the training and test sets are shown in Figure 2A and 2B, respectively.

**Figure 3:**
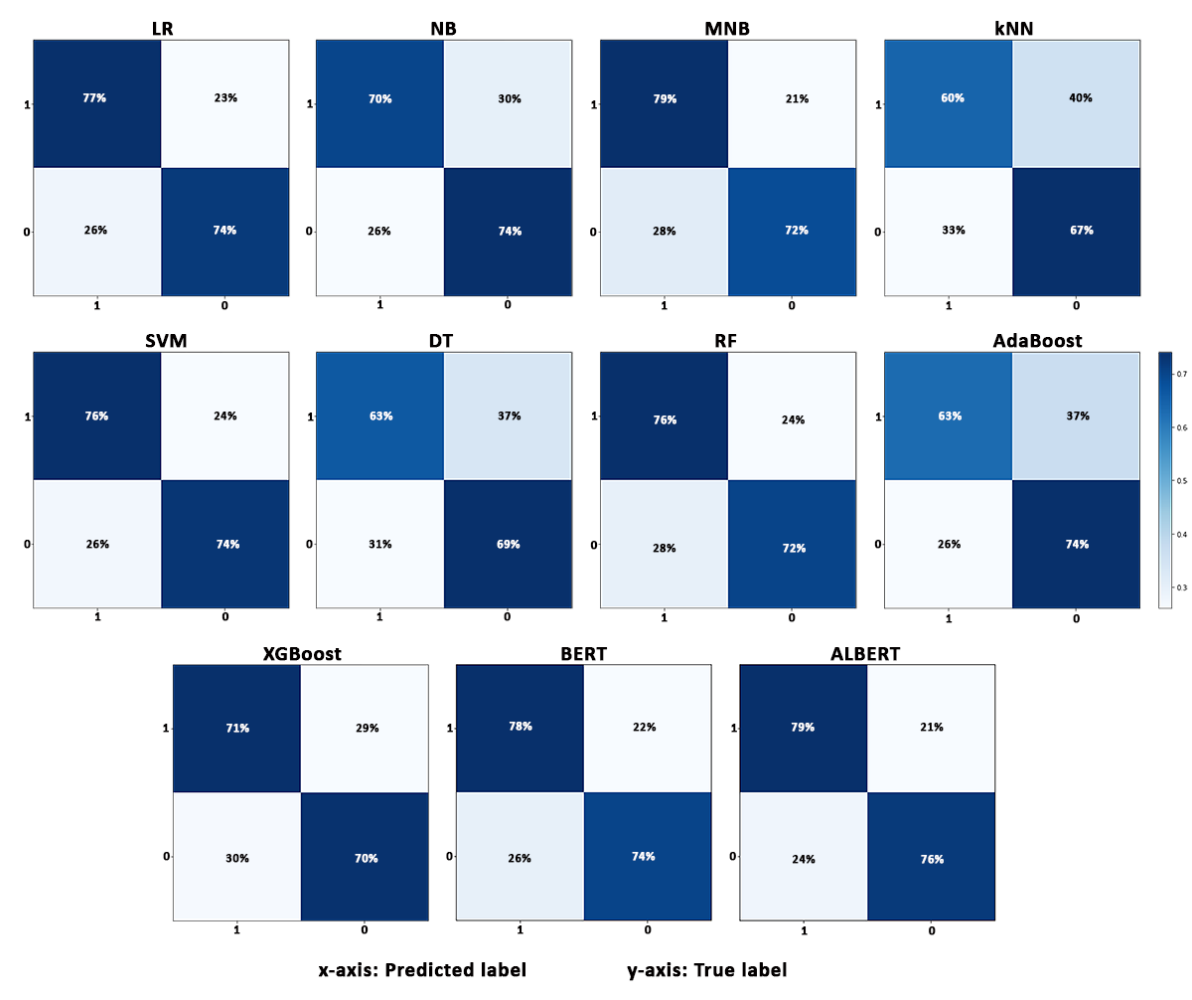
Confusion matrices produced by the competing algorithms for the test set using the First Wave data set. LR: logistics regression; NB: Naïve Bayes; MNB: Multinomial Naïve Bayes; kNN: k-nearest neighbor; SVM: support vector machine; DT: decision tree; RF: random forest; BERT: Bidirectional Encoder Representations from Transformers; ALBERT: A Lite BERT.

Based on the performance of various algorithms on the test set (Table 4), the ALBERT algorithm demonstrated promising performance in class-wise measurements and accurately identifying posts relevant to dementia and COVID-19. Therefore, we concluded that the ALBERT algorithm shows reliable performance for this task. The class-wise results of competing algorithms using confusion matrices are shown in Figure 3.

**Table 3:** The performance of different algorithms for the training set using the First Wave data set.

Algorithm	Mean accuracy % (SD)	Mean precision % (SD)	Mean sensitivity % (SD)	Mean specificity % (SD)	Mean *F*_1_-score % (SD)	Mean AUC^a^ % (SD)
Logistic regression	80.51 (0.18)	68.39 (0.54)	83.88 (0.32)	78.65 (0.19)	75.35 (0.31)	88.96 (0.1)
Naïve Bayes	78.91 (0.29)	62.57 (0.62)	85.07 (0.48)	76.02 (0.27)	72.1 (0.41)	87.86 (0.13)
Multinomial Naïve Bayes	79.3 (0.56)	71.82 (4.46)	79 (2.67)	79.74 (1.82)	75.06 (1.74)	82.23 (1.51)
K-nearest neighbor	78.41 (0.32)	69.78 (2.61)	78.41 (1.79)	78.53 (1.08)	73.78 (0.67)	87.42 (0.23)
Support vector machine	*96.17 (0.13)^b^*	*93.08 (0.23)*	*98.04 (0.22)*	*94.86 (0.15)*	*95.49 (0.15)*	*99.41 (0.05)*
Decision tree	77.39 (0.62)	68.87 (1.48)	76.84 (1.18)	77.76 (0.72)	72.62 (0.87)	86.19 (0.79)
Random forest	88.66 (0.34)	80.83 (1.03)	92.17 (0.42)	86.49 (0.54)	86.12 (0.52)	96.87 (0.09)
AdaBoost	75.08 (0.85)	76.2 (2.34)	69.6 (1.68)	80.21 (0.98)	72.7 (0.74)	83.86 (0.55)
XGBoost	73.99 (0.57)	59.9 (5.67)	75.65 (2.64)	73.41 (1.94)	66.59 (2.57)	82.8 (0.24)
BERT^c^	81.52 (0.1)	75.42 (1.04)	85.13 (1.01)	81.78 (0.2)	80.23 (1.32)	82.98 (0.4)
ALBERT^d^	81.78 (0.45)	74.98 (0.98)	84.08 (0.82)	82.19 (0.61)	79.27 (0.5)	83.13 (0.45)

^a^AUC: area under the curve.

^b^The best values for the performance metrics are in italics.

^c^BERT: Bidirectional Encoder Representations from Transformers.

^d^ALBERT: A Lite BERT.

**Table 4:** The performance of different algorithms for test set using the First Wave data set.

Algorithm	Mean accuracy % (SD)	Mean precision % (SD)	Mean sensitivity % (SD)	Mean specificity % (SD)	Mean *F*_1_-score % (SD)	Mean AUC^a^ % (SD)
Logistic regression	75.13 (0.32)	61.97 (2.81)	76.55 (2.85)	74.39 (2.38)	68.43 (1.96)	82.21 (0.99)
Naïve Bayes	74.26 (0.48)	56.19 (3.39)	78.8 (3.91)	72.29 (2.65)	65.5 (2.65)	82.13 (1.16)
Multinomial Naïve Bayes	72.1 (2.67)	63.6 (4.26)	69.92 (4.52)	73.75 (1.92)	66.43 (2.78)	74.37 (1.87)
K-nearest neighbor	64.31 (1.79)	53.54 (3.98)	60.49 (4.74)	66.94 (1.97)	56.6 (2.5)	73.87 (2.72)
Support vector machine	74.75 (0.22)	62.04 (2.2)	75.65 (2.99)	74.26 (2.1)	68.13 (1.93)	82.09 (1.09)
Decision tree	66.49 (1.18)	56.03 (2.98)	62.97 (3.21)	68.73 (2.4)	59.25 (2.52)	69.64 (2.18)
Random forest	73.37 (0.42)	56.98 (2.4)	75.91 (3.79)	72.15 (2.06)	65.05 (2.46)	80.48 (1.82)
AdaBoost	68.13 (1.68)	67.98 (4.67)	62.44 (3.65)	73.54 (3.04)	64.94 (2.6)	73.67 (1.65)
XGBoost	70.32 (2.64)	54.6 (5.89)	70.75 (3.52)	70.29 (2.88)	61.4 (3.82)	76.82 (1.93)
BERT^b^	81.03 (1.01)	73.87 (3.43)	80.21 (3.1)	77.61 (3.3)	77.87 (2.34)	80.89 (2.3)
ALBERT^c^	*82.92 (0.82)^d^*	*74.55 (8.91)*	*84.83 (5.86)*	*82.24 (5.06)*	*78.81 (4.93)*	*83.53 (3.14)*

^a^AUC: area under the curve.

^b^BERT: Bidirectional Encoder Representations from Transformers.

^c^ALBERT: A Lite BERT.

^d^The best values for the performance metrics are in italics.

#### Study 2

To extend our study to other conditions and evaluate the reliability of the best-performing algorithm in identifying posts relevant to dementia and COVID-19, we applied it to the longitudinal data set. Because this data set shares similarities with the one used in study 1, the results of study 2 can indicate whether the same methods can be used for similar data sets involving mental health disorders, pandemics, and the like. The ALBERT algorithm was used to test the longitudinal data set, and the results, as shown in Table 5, confirmed its effectiveness in correctly identifying posts relevant to dementia and COVID-19 at the second stage of the pandemic.

Although the ALBERT algorithm demonstrated promising performance in study 2, it was important to assess the generalizability and robustness of the methodology introduced by using a similar data set and search terms. Testing an algorithm on a data set with similar words as the training data can result in overfitting and may not generalize well to other data sets. Furthermore, a customized method for a given data set is not overly useful. Accordingly, study 3 was conducted to evaluate the performance of the ALBERT algorithm on a new and unrelated yet adjacent data set.

**Table 5:** The performance of the ALBERT^a^ algorithm using the longitudinal data set.

Algorithm	Mean accuracy %	Mean precision %	Mean sensitivity %	Mean specificity %	Mean *F*_1_-score %	Mean AUC^b^ %
ALBERT	81.23	86.02	79.45	83.41	82.24	81.46

^a^ALBERT: A Lite Bidirectional Encoder Representations from Transformers.

^b^AUC: area under the curve.

#### Study 3

Study 3 aimed to assess the generalizability of the ML algorithm in identifying posts relevant to different mental disorders and pandemics by examining the X discourse on dementia during Alzheimer’s Awareness Month in Canada. Given the variability of health issues and the possibility of different pandemics, it is highly beneficial to have a model that performs well across different contexts. To evaluate the performance of the ALBERT algorithm in this context, we retrained the algorithm using 10% (129/1289 labeled posts) of the Alzheimer’s Awareness Month data set (addressing different mental health disorders and containing different search terms from those in the First Wave and longitudinal data sets) and tested it on the remaining 90% (1160/1289 labeled posts). As shown in Table 6, using the Alzheimer’s Awareness Month data set ALBERT algorithm achieved a reliable and acceptable performance of 80% or higher in all the metrics considered. The results challenged any potential bias of the ALBERT algorithm toward the First Wave and longitudinal data sets and confirmed that it can be applied to identify and classify posts relevant to different mental health disorders during pandemics.

**Table 6:** The performance of the ALBERT^a^ algorithm for training and test sets using the Alzheimer’s Awareness Month data set.

	Mean accuracy %	Mean precision %	Mean sensitivity %	Mean specificity %	Mean *F*_1_-score %	Mean AUC^b^ %
Training set	86.39	89.21	84.98	87.24	82.77	86.24
Test set	80.62	83.40	79.98	80.62	81.65	80.30

^a^ALBERT: A Lite Bidirectional Encoder Representations from Transformers.

^b^AUC: area under the curve.

## Discussion

### Principal Findings

The COVID-19 pandemic has highlighted the needs of vulnerable populations, including patients with dementia and their caregivers. Social media platforms, particularly X, provide a valuable source of data for health care researchers, governments, policy makers, and practitioners to understand the impact of the pandemic on this population. However, the sheer volume and high percentage of irrelevant social media data make it difficult to manually mine, analyze, and identify relevant posts for this purpose.

In this study, we aimed to identify posts relevant to dementia and COVID-19 using a combination of NLP and ML algorithms. Our findings demonstrated that transfer learning algorithms such as ALBERT outperform traditional ML algorithms in identifying relevant posts. The results of study 1 showed the superiority of the ALBERT algorithm and demonstrated that it achieved the best performance for the task of identifying relevant posts. In addition, in study 2, this algorithm demonstrated its capability in identifying posts that share similar content with the data set used in study 1. This indicated that transfer learning algorithms can effectively identify posts relevant to similar disorders for which the algorithm was trained. Furthermore, study 3 revealed a high level of generalizability of these algorithms. This suggests that transfer learning algorithms can be trained for a specific disorder or pandemic and can be applied to different (especially adjacent) disorders or pandemics with comparable performance.

Our study highlights the significant applicability and value of automated approaches in identifying posts relevant to COVID-19 and dementia, and the methodology is applicable to other studies involving the analysis of social media posts. A further application of this study is potential real-time monitoring of public health sentiment during pandemics or other public health crises. Tools can be developed to continuously analyze social media data to track the sentiments, concerns, and information needs of patients with dementia, their caregivers, and the general public. This real-time monitoring can provide invaluable insights to public health authorities, researchers, and policy makers, enabling them to decide about responses, allocate resources efficiently, and provide targeted support to vulnerable populations such as patients with dementia.

### Limitations

Limitations of this study mainly involve the inherent biases in using X data for research. First, X users represent a particular portion of the population, often more tech-savvy, and this bias can result in the underrepresentation of older or less internet-connected demographics. In addition, X data may not capture the perspectives of individuals who do not use this platform for discussing health-related topics, potentially leading to an inaccurate perspective on public sentiment. Furthermore, linguistic and cultural biases can exist in the language and topics discussed on X, and these biases should also be considered when interpreting the findings. Moreover, this study focused on posts filtered by the keywords “Coronavirus” and “dementia.” Although the methods and analysis were tested on different data sets, they are particularly relevant to data related to COVID-19 and dementia. To increase generalizability, future research could explore the use of various other search keywords. Another limitation is the unexplored possible effect of post length on algorithm performance. Because the study did not consider post length, future research could include sensitivity analysis to determine its impact on algorithm performance. Finally, although posts reflect people’s thoughts at a specific moment, textual features alone may not fully reflect overall sentiments. Various syntactic and semantic post features could be explored to enhance algorithm performance. These limitations do not undermine this study’s contributions.

### Conclusions

The COVID-19 pandemic has created a pressing need for policy makers to make timely decisions to address the needs of vulnerable populations, such as patients with dementia and their caregivers. Social media platforms, particularly X, offer a wealth of data that can provide invaluable insights into COVID-19 and its impact on patients with dementia and other vulnerable populations. However, the sheer volume and high fraction of irrelevant data make it difficult to extract and analyze relevant content. An automated tool that can accurately identify posts relevant to a given topic would be of great value to health care researchers, social scientists, policy makers, and practitioners in analyzing such posts.

In this study, we explored the use of an automated approach to identify posts relevant to dementia and COVID-19 using various ML algorithms. Our study shows that transfer learning algorithms outperform traditional ML algorithms, with the ALBERT algorithm achieving the best performance among the algorithms tested. The reliability of the results was confirmed using independent data sets, highlighting the ability of these algorithms to be part of an automated methodology for identifying posts relevant to dementia and COVID-19. Such a methodology can also be applied to help facilitate other studies that involve analysis of social media posts, and it can aid in effective and timely decision-making during times of acute need.

Future research could include the analysis of multiple features of posts to further increase identification performance and the reliability of the algorithms. In addition, as pandemic or other communicable disease-related data continue to become available on social media, the development of a range of analytics and ML-based solutions based on processing such data can lead to enhanced support for patients and their caregivers. Other future research directions include use of specific medical-based models to improve performance, application of the approach to nonmedical data to test its generality, and incorporation of cross-language recognition to gain insight from other cultures.

## Appendix

Multimedia Appendix 1 The word cloud of the most frequently used word in each data set.

## Abbreviations

ALBERT: A lite BERT
AUC: area under the curve
BERT: Bidirectional Encoder Representations from Transformers
CV: cross-validation
DT: decision tree
kNN: k-nearest neighbor
LR: logistics regression
ML: machine learning
MNB: multinomial naïve Bayes
NB: naïve Bayes
NLP: natural language processing
RF: random forest
SVM: support vector machine
TF-IDF: term frequency–inverse document frequency
XGBoost: Extreme Gradient Boosting

## Data Availability

The data sets generated or analyzed during this study are available from the corresponding author on reasonable request.
