# Supplementary material for: Identifying X (Formerly Twitter) Posts Relevant to Dementia and COVID-19: Machine Learning Approach
Source: JMIR Form Res. 2024 Jun 4;8:e49562. doi: 10.2196/49562 (PMC11185906; doi:10.2196/49562)
Supplement: Multimedia Appendix 1 [file formative_v8i1e49562_app1.docx]

Multimedia Appendix

Identifying X (Formerly Twitter) Posts Relevant to Dementia and COVID-19: Machine Learning Approach


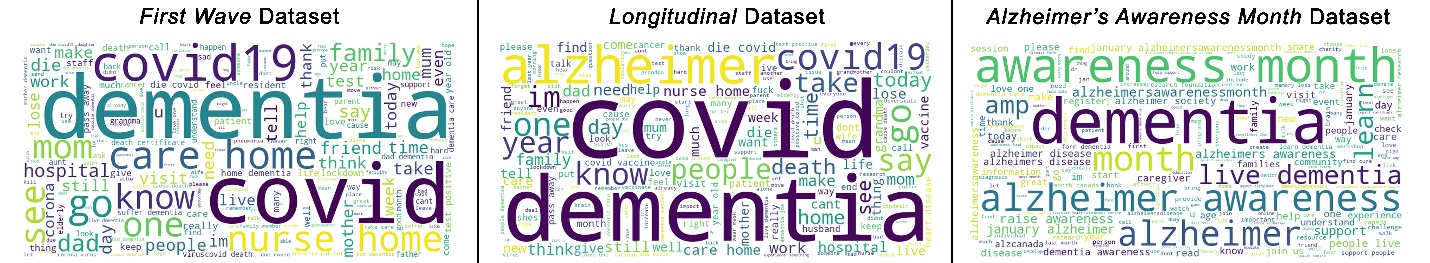


Figure S1: The word cloud of the most frequently used word in each data set.
